# Risk Factors for Infection-Attributable Mortality in Patients With *Staphylococcus aureus* Bacteremia: A Competing Risk Analysis

**DOI:** 10.1093/ofid/ofae734

**Published:** 2024-12-24

**Authors:** Seongman Bae, Min Soo Kook, Euijin Chang, Jiwon Jung, Min Jae Kim, Yong Pil Chong, Sung-Han Kim, Sang-Ho Choi, Sang-Oh Lee, Yang Soo Kim

**Affiliations:** Division of Infectious Diseases, Department of Internal Medicine, Asan Medical Center, University of Ulsan College of Medicine, Seoul, Republic of Korea; Center for Antimicrobial Resistance and Microbial Genetics, University of Ulsan College of Medicine, Seoul, Republic of Korea; Division of Infectious Diseases, Department of Internal Medicine, Asan Medical Center, University of Ulsan College of Medicine, Seoul, Republic of Korea; Division of Infectious Diseases, Department of Internal Medicine, Asan Medical Center, University of Ulsan College of Medicine, Seoul, Republic of Korea; Division of Infectious Diseases, Department of Internal Medicine, Asan Medical Center, University of Ulsan College of Medicine, Seoul, Republic of Korea; Division of Infectious Diseases, Department of Internal Medicine, Asan Medical Center, University of Ulsan College of Medicine, Seoul, Republic of Korea; Division of Infectious Diseases, Department of Internal Medicine, Asan Medical Center, University of Ulsan College of Medicine, Seoul, Republic of Korea; Division of Infectious Diseases, Department of Internal Medicine, Asan Medical Center, University of Ulsan College of Medicine, Seoul, Republic of Korea; Division of Infectious Diseases, Department of Internal Medicine, Asan Medical Center, University of Ulsan College of Medicine, Seoul, Republic of Korea; Division of Infectious Diseases, Department of Internal Medicine, Asan Medical Center, University of Ulsan College of Medicine, Seoul, Republic of Korea; Division of Infectious Diseases, Department of Internal Medicine, Asan Medical Center, University of Ulsan College of Medicine, Seoul, Republic of Korea; Center for Antimicrobial Resistance and Microbial Genetics, University of Ulsan College of Medicine, Seoul, Republic of Korea

**Keywords:** attributable death, competing risk analysis, risk factor, *Staphylococcus aureus*, *S. aureus* bacteremia

## Abstract

**Background:**

Identifying risk factors for mortality in patients with *Staphylococcus aureus* bacteremia (SAB) is crucial due to its high fatality. However, data on risk factors for infection-attributable deaths considering competing risk events such as non-infection-attributable deaths remain limited. We performed a competing risk analysis to elucidate risk factors associated with 30-day infection-attributable mortality in a large cohort of patients with SAB.

**Methods:**

This retrospective cohort study included adult patients diagnosed with SAB at a tertiary hospital from August 2008 to December 2019. Competing risk analysis was performed using Fine and Gray models to estimate subdistribution hazard ratios (sHRs) for 30-day infection-attributable death.

**Results:**

Among 1936 patients, 444 (22.9%) died within 30 days. Of these, 338 (76.1%) were infection-attributable and 106 (23.9%) were non-infection-attributable deaths. The multivariable Fine and Gray model identified significant risk factors for 30-day infection-attributable death (sHRs with 95% confidence intervals): an increase in age by 10 years (1.14 [1.02–1.26]), presence of malignancy (1.54 [1.17–2.02]), liver cirrhosis (2.15 [1.56–2.97]), corticosteroid use (1.61 [1.19–2.17]), septic shock (3.28 [1.98–5.42]), elevated C-reactive protein (1.60 [1.19–2.14]), pneumonia (1.81 [1.21–2.72]), persistent bacteremia (1.73 [1.31–2.30]), and failure to remove the eradicable focus (2.40 [1.38–4.19]) or absence of an eradicable focus (1.49 [1.08–2.04]). Except for age and malignancy, these factors were not significantly associated with non-infection-related death.

**Conclusions:**

Specific risk factors for infection-attributable death in patients with SAB were identified, distinct from those for nonattributable death. These findings can aid in the early identification of patients at risk for SAB-attributable mortality.

## Graphical Abstract

**Graphical Abstract:**
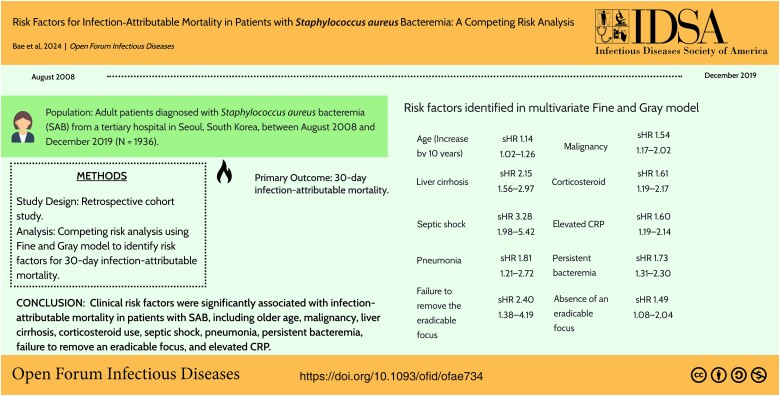
This graphical abstract is also available at Tidbit: https://tidbitapp.io/tidbits/risk-factors-for-infection-attributable-mortality-in-patients-with-staphylococcus-aureus-bacteremia-a-competing-risk-analysis?utm_campaign=tidbitlinkshare&amp;utm_source=IO

*Staphylococcus aureus* is the leading bacterial cause of infection-attributable mortality worldwide [1]. Due to its virulence and antimicrobial resistance, *S aureus* remains a significant public health burden with a high mortality rate despite antibiotic therapy [2]. Several studies have been conducted on the risk factors for mortality from *S aureus* infections, and clinical variables such as age, endocarditis, and persistent bacteremia have been regarded as classic mortality risk factors [3, 4]. However, most of the previous studies did not differentiate between infection-attributable mortality and non-infection-attributable mortality, and there was potential for the risk variables to be overestimated by not considering competing events such as non-infection-attributable mortality. In this study, we aimed to elucidate the risk factors for infection-attributable mortality by conducting a competing risk analysis in patients with *S aureus* bacteremia (SAB) over an 11-year period.

## METHODS

### Study Design, Settings, and Population

This is a retrospective cohort study using data from a large cohort of patients diagnosed with SAB. In this cohort, all adult patients with SAB admitted to a 2700-bed tertiary hospital (Asan Medical Center, Seoul, South Korea) were consecutively enrolled from March 2007 to December 2019. We only included patients with a first episode of bacteremia. The exclusion criteria were as follows: (*i*) age <18 years; (*ii*) patient discharged at the emergency department; (*iii*) different organisms isolated from the same blood sample; (*iv*) previous history of *S aureus* bacteremia within 90 days; (*v*) referred from other hospitals after treating SAB for ≥3 days; or (*vi*) identification in outpatient clinics. All patients included in this study received consultations from infectious disease (ID) experts through the automated consult system during their SAB treatment. Clinical information, including demographics, infection foci, results of tests performed to evaluate metastatic infections, and treatment, was reviewed by 2 or more ID experts 1 week after the day of the first positive culture. Subsequently, clinical outcomes, including bacteremia duration, recurrence, mortality, and metastatic infection were reviewed again by chart review of electronic health records up to 90 days after the day of the first positive culture. The primary outcome of this study was infection-attributable mortality within 30 days, because 30-day mortality can capture most infection-attributable deaths while minimizing the noise from non-infection-related deaths that increase after 30 days [5]. This study was conducted in accordance with the STROBE (Strengthening the Reporting of Observational Studies in Epidemiology) guidelines for observational studies [6].

### Ethics

This study was approved by the institutional review board (IRB) of Asan Medical Center (IRB number 2013-0234). Informed consent was waived because of the retrospective nature of the study and the fact that the patient identifiers were encrypted before analysis.

### Bacterial Strains, Culture, and Genotyping

*Staphylococcus aureus* isolates were identified using standard methods, and antimicrobial susceptibility was determined in accordance with standard criteria based on the MicroScan system (Dade Behring, West Sacramento, California) and the Clinical and Laboratory Standards Institute (CLSI). Methicillin-resistant *S aureus* (MRSA) was confirmed based on the minimum inhibitory concentration (MIC) of oxacillin and the presence of the *mecA* gene. *Staphylococcus aureus* isolates present in the blood were collected, stored, and subcultured for genotyping; the genotyping included multilocus sequence typing (MLST) [7]. The functionality of the accessory gene regulator (*agr*) operon was investigated using the δ-hemolysin test as previously described [8]. The MIC of vancomycin was determined using the broth microdilution method in accordance with CLSI guidelines [9].

### Definitions

The index date was defined as the day of the first positive blood culture for *S aureus*. The observation period was defined as the interval from the index date to the time of death or the end of follow-up. Mortality was assessed for 90 days after the index date and categorized into 3 classifications [10]: first, “definite” infection-attributable death was defined as death directly resulting from complications of an infection or occurring amid persistent symptoms and signs of an infection; second, non-infection-attributable death was defined as a patient surviving until the completion of antibiotic treatment or died due to other causes in the absence of pronounced symptoms of infection; third, where neither the attributable nor nonattributable criteria were met, the death was categorized as a “possible” attributable death. In this study, infection-attributable death was defined as including both definite and possible attributable deaths. Attributable mortality was adjudicated by at least 2 ID specialists independently, and in cases of disagreement, a consensus was reached through further discussion. Individuals who were lost to follow-up without any events before day 90 were right-censored on the last day of admission or visit.

The mode of acquisition for SAB was categorized as community-acquired, healthcare-associated, or hospital-acquired, as described elsewhere [11]. Corticosteroid use was defined as active use of corticosteroids at the time of SAB, regardless of the dose. Immunosuppressive agent use was defined as the use of immunosuppressing medication at the time of SAB, with a minimum duration of 1 week prior to the SAB. Neutropenia was defined as an absolute neutrophil count of <500 cells/μL at the time of SAB. The main focus of infection was determined by 2 or more ID experts 1 week after the first positive *S aureus* blood culture, considering the initial symptoms, clinical course, imaging studies, and echocardiography results. In cases where multiple concurrent foci were present, the decision was based on factors such as the onset of symptoms, time of detection, life-threatening potential, bacterial burden, and the expected duration of treatment for each specific focus. A central venous catheter (CVC)–related bloodstream infection, according to the Infectious Diseases Society of America criteria, is established if either of the following conditions is met: (*i*) a semi-quantitative culture of the removed catheter tip shows >15 colony-forming units using the roll plate technique, and the same organism is isolated from both the catheter tip and peripheral blood; or (*ii*) a differential time to positivity of >2 hours is indicative [12]. Additionally, CVC-related bloodstream infection is considered if the patient has a catheter and at least 1 positive blood culture for *S aureus*, accompanied by a compatible clinical presentation and the absence of any other identifiable source of infection. Peripheral venous catheter–related bloodstream infection was defined when visible thrombophlebitis at a peripheral intravenous catheter insertion site was documented at the time of bacteremia and no other definite source of bacteremia was identified [13]. A diagnosis of pneumonia was made when pulmonary infiltrates were documented and the same organism causing the bacteremia was isolated from the respiratory specimens [14]. Infective endocarditis was defined using the modified Duke criteria [15]. Severity of infection was assessed using the presence of sepsis or septic shock at the time of bacteremia. C-reactive protein (CRP) was dichotomized into a binary variable using a threshold of 10 mg/dL, measured at the time of SAB or within 48 hours of the initial positive blood culture, as in our previous study [16]. MLST types were classified with sequence type (ST) 5 being the most prevalent hospital-associated clone in South Korea, ST72 as the most prevalent community-associated clone in South Korea, and others [17, 18]. Persistent bacteremia was defined as blood culture(s) remaining positive for ≥3 days despite appropriate antibiotic therapy [19]. Appropriate antibiotic therapy was defined as the use of an effective intravenous agent against *S aureus*, as the efficacy of oral antibiotics has not been validated for initial treatment for SAB. For MRSA, appropriate antibiotics included vancomycin, teicoplanin, and linezolid. For methicillin-susceptible *S aureus* (MSSA), anti-staphylococcal β-lactams, such as cefazolin and nafcillin, as well as vancomycin, teicoplanin, and linezolid, were included as appropriate. Additionally, for MSSA, ampicillin-sulbactam, piperacillin-tazobactam, ceftriaxone, and cefepime were also regarded as having sufficient anti-staphylococcal activity. Focus removal was categorized into 3 groups based on the presence of an eradicable infection focus and whether surgical or interventional procedures for source control were performed: complete focus removal, incomplete focus removal, and no eradicable focus.

### Statistical Analysis

Categorical variables were analyzed using χ^2^ or Fisher exact test, whereas continuous variables were analyzed using Student *t* test. To reduce the number of 80 clinical and microbiological variables, hierarchical variable clustering was performed, and the number of variables was reduced to those more representative within the same domain. A competing risk analysis was conducted to estimate the subdistribution hazard ratio (sHR) and its 95% confidence interval (CI) for 30-day infection-attributable mortality using a Fine and Gray model [20]. To find risk factor variables associated with attributable deaths, a univariate Fine and Gray regression model was performed, and only variables showing a *P* value <.10 were included in the multivariable model. For the final multivariable model, a stepwise variable selection was conducted by using backward elimination according to the Akaike information criterion. Multi-collinearities were checked by the variance inflation factor. The assumption of proportionality was checked using Schoenfeld residuals. For the internal validation process, the bootstrap-corrected CIs for variables included in the final multivariable model were calculated [21]. We conducted several sensitivity analyses on the risk factors included in the final model. First, we performed an analysis including only “definite” attributable death cases as the outcome, rather than both “definite” and “possible” cases in the primary analysis. Second, we analyzed 90-day infection-attributable death rather than 30-day infection-attributable death as the outcome. Third, we performed a landmark analysis, setting day 3 as the index day to adjust for potential bias by defining persistent bacteremia as a positive blood culture persisting for ≥3 days, thus excluding cases who died before persistent bacteremia could be diagnosed. Fourth, we performed a cause-specific hazard analysis using a Cox proportional hazards model as a sensitivity analysis to provide direct etiological relationships between risk factors and mortality. Subgroup analyses were performed based on age group (≥60 years or <60 years), sex (male or female), and mode of acquisition (community-acquired or non-community-acquired). All reported *P* values were 2-sided, and a *P* value <.05 was considered statistically significant. Data manipulation and statistical analyses were conducted using R software, version 4.0.4.

## RESULTS

**Figure 1.**
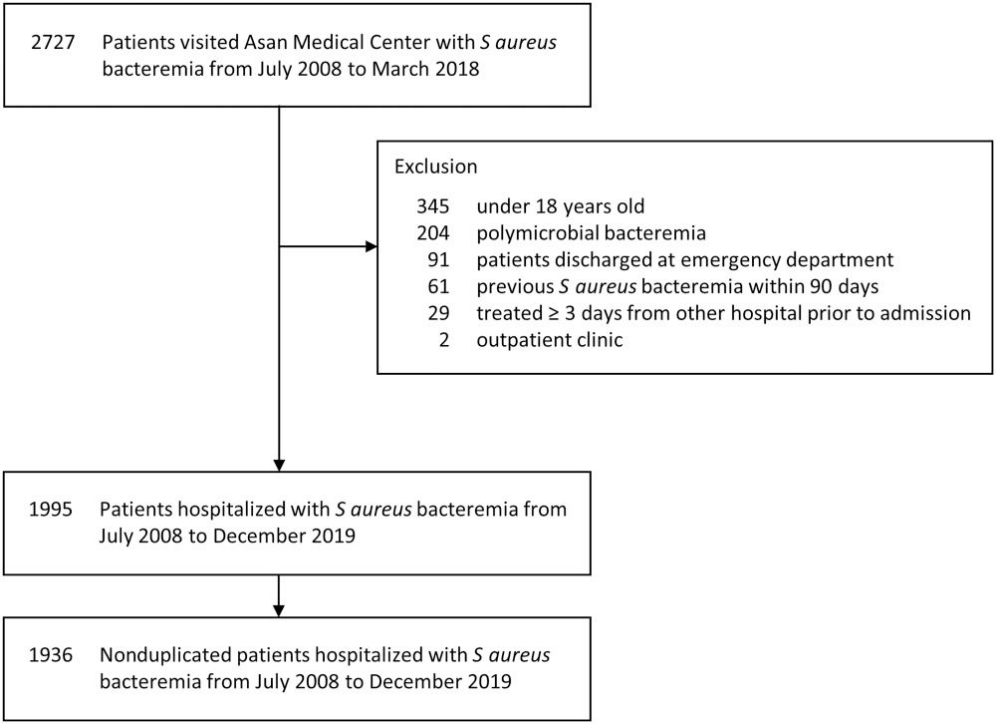
Flowchart of the study population.

**Figure 2.**
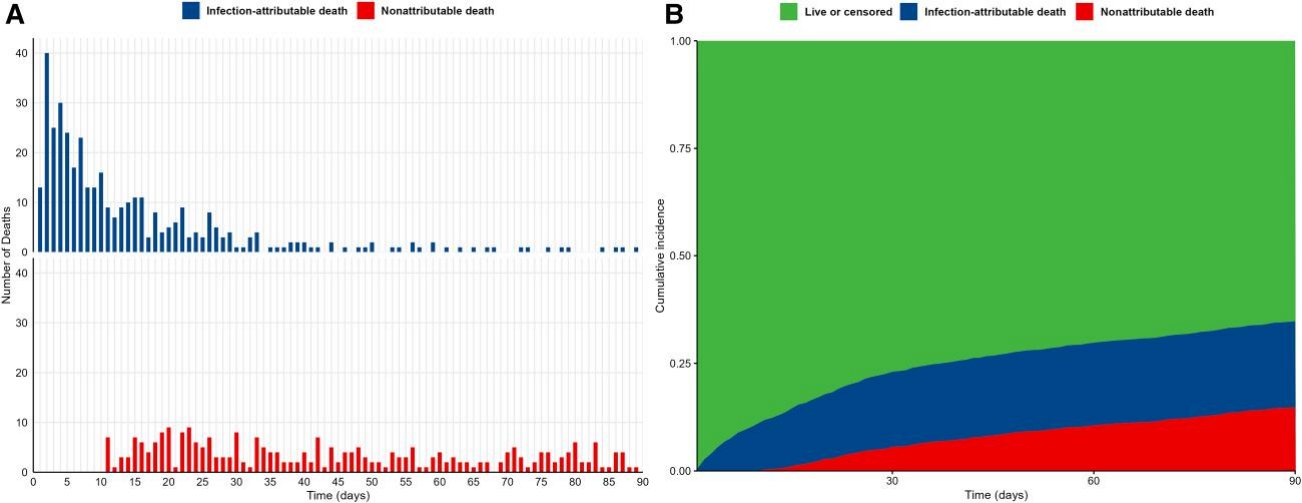
Infection-attributable deaths and infection-nonattributable deaths during 90 days among patients with *Staphylococcus aureus* bacteremia. *A*, Number of infection-attributable deaths and infection-nonattributable deaths per day during the 90-day period. *B*, Cumulative incidence for infection-attributable deaths and infection-nonattributable deaths.

A total of 1936 nonduplicated patients with SAB were included in the study (Figure 1). Of 1936 patients with SAB, 1876 (96.9%) patients either completed the 90-day follow-up or experienced death, while the remaining 60 (3.1%) were lost to follow-up without death before day 90. The characteristics of the patients with SAB are summarized in Table 1. Their median age was 63 years, 62.7% were male, and 51.7% had infections caused by MRSA (Table 1). The microbiological characteristics of the *S aureus* isolates, including vancomycin MIC, MLST type, and *agr* dysfunctional status, have been summarized in Supplementary Table 1. Among the 1936 patients, 444 (22.9%) died within 30 days, with 338 (76.1%) deaths being infection-attributable and 106 (23.9%) being non-infection-attributable. The characteristics of the patients who experienced attributable death, non-attributable death, and those who survived within the 30 days are summarized in Supplementary Table 2. Over the 90-day period, a total of 665 (34.3%) patients died, with 385 (57.9%) attributable to infection and 280 (42.1%) nonattributable to infection. Of the 385 cases of attributable deaths within 90 days, 337 (87.5%) occurred within 30 days (Figure 2).

**Table 1.** Baseline Characteristics of the Study Group

Characteristic	Patients With SAB (N = 1936)
Age, y, median (IQR)	63 (53–72)
Male sex	1214 (62.7)
MRSA	1000 (51.7)
Mode of acquisition	
Community acquired	282 (14.6)
Healthcare associated	600 (31.0)
Nosocomial	1054 (54.4)
Comorbidities	
Malignancy	932 (48.1)
Diabetes mellitus	592 (30.6)
Hypertension	789 (40.8)
Chronic kidney disease	275 (14.2)
Liver cirrhosis	306 (15.8)
Chronic lung disease	55 (2.8)
Ischemic heart disease	173 (8.9)
CCI score, median (IQR)	3 (2–5)
Predisposing factors	
Neutropenia	112 (5.8)
Immunosuppressant agent	132 (6.8)
Corticosteroid use	477 (24.6)
Indwelling prosthetic devices	
Central venous catheter	760 (39.3)
Cardiac implantable electronic device	22 (1.1)
Prosthetic heart valve	63 (3.3)
Vascular graft	141 (7.3)
Orthopedic implant	72 (3.7)
Severity of infection	
No sepsis	356 (18.4)
Sepsis	1308 (67.6)
Septic shock	272 (14.0)
Elevated CRP (≥10 mg/dL)	934 (48.2)
Main focus of infection	
CVC-related	494 (25.5)
Peripheral catheter–related	127 (6.6)
Pneumonia	195 (10.1)
Skin and soft tissue infection	171 (8.8)
Surgical wound infection	107 (5.5)
Endocarditis	72 (3.7)
Bone and joint infection	161 (8.3)
Primary bacteremia	334 (17.3)
Others	277 (14.3)
Length of bacteremia, d, median (IQR)	1 (0–3)
Persistent bacteremia (≥3 d)	504 (28.1)
Focus removal	
Complete	866 (44.7)
Not complete	112 (5.8)
No eradicable focus	958 (49.5)
Time to appropriate antibiotic therapy, d, median (IQR)	0 (0–1)
Length of antibiotic therapy, d, median (IQR)	19 (12–33.5)
Type of antibiotic therapy	
Anti-staphylococcal β-lactams	692 (35.7)
Glycopeptides	915 (47.3)
Others	329 (17.0)
All-cause mortality	
14-d	264 (13.6)
30-d	444 (22.9)
90-d	665 (34.3)

Data are presented as No. (%) unless otherwise indicated.

Abbreviations: CCI, Charlson comorbidity index; CRP, C-reactive protein; CVC, central venous catheter; IQR, interquartile range; SAB, *Staphylococcus aureus* bacteremia.

### Factors Associated With Infection-Attributable Death in Patients With *S aureus* Bacteremia

In univariate Fine and Gray regression analysis, the following variables demonstrated a significant association with attributable death: an increase in age by 10 years (sHR, 1.17 [95% CI, 1.08–1.26]), malignancy (sHR, 1.57 [95% CI, 1.27–1.95]), diabetes (sHR, 0.76 [95% CI, .59–.97]), liver cirrhosis (sHR, 1.49 [95% CI, 1.15–1.93]), corticosteroid use (sHR, 1.45 [95% CI, 1.16–1.83]), sepsis (sHR, 1.63 [95% CI, 1.12–2.36]), septic shock (sHR, 6.34 [95% CI, 4.29–9.37]), elevated CRP (sHR, 2.00 [95% CI, 1.61–2.50]), CVC-related infection (sHR, 0.69 [95% CI, .53–.90]), pneumonia (sHR, 2.89 [95% CI, 2.22–3.75]), skin and soft tissue infection (sHR, 0.51 [95% CI, .32–.83]), primary bacteremia (sHR, 1.48 [95% CI, 1.14–1.91]), persistent bacteremia (sHR, 1.81 [95% CI, 1.38–2.37]), and failure to remove the infection focus (sHR, 2.81 [95% CI, 1.84–4.31]) or the absence of an eradicable infection focus (sHR, 2.59 [95% CI, 2.03–3.31]) (Supplementary Table 3).

**Figure 3.**
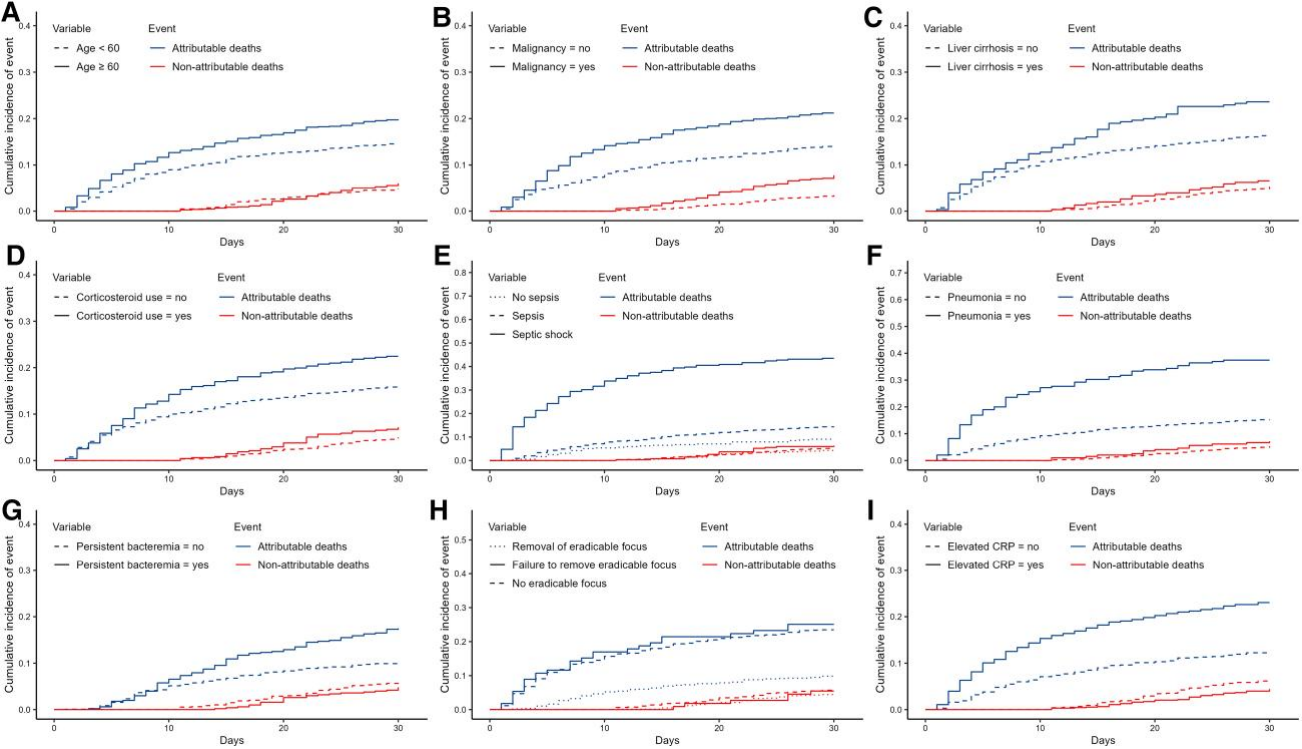
Cumulative incidence plots of infection-attributable deaths and the competing risk event (infection-nonattributable deaths) by variable. *A*, Age group. The age groups in 10-year intervals were simplified into 2 categories, ≥60 years and <60 years. *B*, Malignancy. *C*, Liver cirrhosis. *D*, Corticosteroid use. *E*, Presence of sepsis or septic shock. *F*, Pneumonia. *G*, Persistent bacteremia. *H*, Removal of eradicable focus. *I*, Elevated serum C-reactive protein (CRP) level.

In the final multivariable model, the following variables were included: age, malignancy, liver cirrhosis, corticosteroid use, septic shock, elevated CRP, pneumonia, persistent bacteremia, and focus removal status (categorized as complete, incomplete, or absence of an eradicable focus). Significant associations with 30-day infection-attributable death were observed for the following variables: age increasing by 10 years (sHR, 1.14 [95% CI, 1.02–1.26]; *P* = .02), malignancy (sHR, 1.54 [95% CI, 1.17–2.02]; *P* = .002), liver cirrhosis (sHR, 2.15 [95% CI, 1.56–2.97]; *P* < .001), corticosteroid use (sHR, 1.61 [95% CI, 1.19–2.17]; *P* = .002), septic shock (sHR, 3.28 [95% CI, 1.98–5.42]; *P* < .001), elevated CRP (sHR, 1.60 [95% CI, 1.19–2.14]; *P* = .002), pneumonia (sHR, 1.81 [95% CI, 1.21–2.72]; *P* = .004), persistent bacteremia (sHR, 1.73 [95% CI, 1.31–2.30]; *P* < .001), and failure to remove the eradicable focus (sHR, 2.40 [95% CI, 1.38–4.19]; *P* = .002) or the absence of an eradicable focus (sHR, 1.49 [95% CI, 1.08–2.04]; *P* = .01) (Table 2 and Figure 3). The proportional hazards assumption for each variable was checked by examining Schoenfeld residuals, and no significant violations were found (Supplementary Figure 1). The final model had a C-index of 0.75, and the calibration plot of the final model is presented in the Supplementary Figure 2. In the internal validation of the final multivariable model, the bootstrapped corrected sHRs and CIs for variables associated with attributable death were robustly observed as the significant risk factors (Supplementary Table 4). The results were robust in the sensitivity analyses for variables associated with attributable death in the final multivariable model (Supplementary Tables 5–8). In the subgroup analyses, there were a few instances where the *P* for interaction was significant; however, the 95% CIs for the subgroups overlapped, and no significant changes in the direction of the hazard ratios were found (Supplementary Tables 9).

**Table 2.** Results of Multivariable Fine and Gray Model for Risk Factors of 30-Day Attributable Death in Patients With *Staphylococcus aureus* Bacteremia

Variable	Infection-Attributable Death			Nonattributable Death		
	sHR	(95% CI)	*P* Value	sHR	(95% CI)	*P* Value
Age (per 10-y increase in age)^a^	1.14	(1.02–1.26)	.**02**	1.17	(1.02–1.34)	.**02**
Malignancy	1.54	(1.17–2.02)	.**002**	2.19	(1.42–3.36)	**<**.**001**
Liver cirrhosis	2.15	(1.56–2.97)	**<**.**001**	1.16	(.69–1.96)	.58
Corticosteroid use	1.61	(1.19–2.17)	.**002**	1.38	(.90–2.10)	.14
Severity of infection			** **			
No sepsis	(reference)		** **	(reference)		
Sepsis	1.33	(.86–2.05)	.20	1.23	(.70–2.15)	.47
Septic shock	3.28	(1.98–5.42)	**<**.**001**	1.82	(.89–3.69)	.10
Pneumonia	1.81	(1.21–2.72)	.**004**	1.33	(.71–2.47)	.37
Persistent bacteremia	1.73	(1.31–2.30)	**<**.**001**	0.91	(.57–1.45)	.68
Focus removal			** **			
Complete	(reference)		** **	(reference)		
Not complete	2.40	(1.38–4.19)	.**002**	1.18	(.47–2.97)	.72
No eradicable focus	1.49	(1.08–2.04)	.**01**	1.32	(.86–2.01)	.20
C-reactive protein ≥10 mg/dL	1.60	(1.19–2.14)	.**002**	0.66	(.42–1.02)	.06

Significant *P* values are indicated in bold.

Abbreviations: CI, confidence interval; sHR, subdistribution hazard ratio.

^a^Age was analyzed using 10-year intervals.

## DISCUSSION

In this study, we used a competing risk regression model to identify risk factors associated with infection-attributable mortality in patients with SAB from a large observational cohort. The identified risk factors for infection-attributable deaths included older age, malignancy, liver cirrhosis, corticosteroid use, septic shock, pneumonia, persistent bacteremia, failure to remove an eradicable focus, and elevated CRP. Notably, all identified risk factors for attributable death, except for increasing age and malignancy, showed no significant association with nonattributable death. This indicates a distinct risk factor profile for attributable death, differing from that of nonattributable death. Therefore, these findings can contribute to the early identification of populations at risk for infection-attributable mortality in patients with SAB.

We found that clinical variables such as age, malignancy, liver cirrhosis, corticosteroid use, septic shock, persistent bacteremia, pneumonia, and source control were significantly associated with attributable death in patients with SAB, which were consistently reported as mortality risk factors in SAB in previous studies [22–25]. Conversely, microbiological variables such as vancomycin MIC, MLST type, and *agr* dysfunction, which have been controversial in terms of their potential impact on SAB mortality, were not found to have a significant association with attributable death in our study [26–28]. Taken together, attributable deaths in patients with SAB were associated with various risk factors, primarily depending on clinical variables rather than microbiological ones. Interestingly, age and malignancy were more strongly associated with non-infection-attributable death compared to infection-attributable death, suggesting that these variables may represent an overall frailty or vulnerability in patients rather than being direct predictors of infection-related mortality. This differential impact underscores the need for careful interpretation of these variables when considering risk factors for infection-attributable death. Future studies may need to address these factors more critically, possibly through competing risk analysis, to better delineate their roles in predicting different types of mortality outcomes in SAB. In addition, the interpretation of the lack of a significant association between microbiological variables and mortality in our study requires caution. This is because these variables were evaluated only in a univariate model and were not included in the final multivariable model, potentially limiting the comprehensiveness of our findings.

We found a 90-day mortality rate of 34.3% among SAB patients, with 57.9% of deaths attributable to infection. Notably, the majority of these attributable deaths (87.5%) occurred within the first 30 days. This pattern closely mirrors findings from a previous multicenter prospective study in the Netherlands, which involved 490 SAB patients and reported a 33% 90-day mortality rate, with 60% due to infection-related causes and 90% of these occurring within the first 30 days, emphasizing the consistency in the timing and proportion of attributable deaths across studies [10]. Given the repeated confirmation that the majority of attributable mortality occurs within 30 days, it becomes evident that 30-day attributable mortality represents a significant outcome indicator for future research and clinical trials of SAB patients.

There were several limitations of this study. First, due to its observational nature and the fact that it was a single-center SAB cohort study, its generalizability is limited. Second, while the differences between infection-attributable and nonattributable related deaths have been reported, the definition of attributable death varies and it remains challenging to clearly define attributable and nonattributable deaths [5, 29]. Despite this ambiguity in defining attributable mortality, we found that the results of our primary analysis, which included both definite and possible cases, were consistent with the sensitivity analysis that included only definite cases. Additionally, most variables associated with infection-attributable death did not show significant associations with nonattributable death, suggesting the presence of specific risk factors unique to infection-attributable death. Third, the risk factors for non-infection-attributable death derived in this study require further validation. Since our analysis focused on variables relevant to SAB, we may have missed variables important for non-infection-attributable death. This could result in unmeasured confounding, as the mechanisms underlying non-infection-attributable death may differ from those of infection-attributable death. Additionally, although some deaths were classified as non-infection-attributable in this study, it is difficult to clearly define them as such since these deaths might have been avoidable if SAB had not occurred. Further studies designed to include uninfected patients as a control group are needed to validate the results for non-infection-attributable deaths. Fourth, microbiological variables such as various virulence factor genes of *S aureus* were not included in the analysis, although no significant association was found between virulence genes and mortality in SAB from a previous study [30]. Notably, further research is needed to elucidate the impact of Panton-Valentine leukocidin (PVL) on clinical outcomes in patients with SAB, given that PVL is generally absent in *S aureus* strains in Korea [17, 31]. Fifth, the CRP cutoff used in this study (CRP ≥10 mg/dL) was adopted from prior literature where it showed significance in relation to persistent bacteremia. However, it is possible that a more optimal cutoff could exist for predicting different clinical outcomes, such as mortality, which may limit the accuracy of using this specific threshold across various contexts.

In conclusion, this study identifies several clinical risk factors significantly associated with infection-attributable mortality in patients with SAB, including older age, malignancy, liver cirrhosis, corticosteroid use, septic shock, pneumonia, persistent bacteremia, failure to remove an eradicable focus, and elevated CRP. The robustness of our results in sensitivity analyses and the distinct risk factor profiles for infection-attributable and nonattributable deaths suggest that these clinical variables play a crucial role in patient outcomes. These clinical variables can aid in the early identification and intervention for patients with SAB who are at high risk for infection-attributable mortality.

## Supplementary Material

ofae734_Supplementary_Data

## Supplementary Data

Supplementary materials are available at *Open Forum Infectious Diseases* online. Consisting of data provided by the authors to benefit the reader, the posted materials are not copyedited and are the sole responsibility of the authors, so questions or comments should be addressed to the corresponding author.

## Notes

***Disclaimer.*** The funders had no role in the study design, data collection and analysis, decision to publish, or preparation of the manuscript.

***Financial support*.** This research was supported by the Korea National Institute of Health research project (grant number 2022-ER2005-00 to Y. S. K.) and the Asan Institute for Life Sciences, Asan Medical Center, Seoul, Korea (grant number 2024IF0018-1 to S. B.).
