# Supplementary material for: Risk Factors for Infection-Attributable Mortality in Patients With Staphylococcus aureus Bacteremia: A Competing Risk Analysis
Source: Open Forum Infect Dis. 2024 Dec 24;12(1):ofae734. doi: 10.1093/ofid/ofae734 (PMC11704955; doi:10.1093/ofid/ofae734)

**Table of Contents**

| **Supplementary Table 1. Microbiological characteristics of *S. aureus* isolated from patients** | Page 2 |
| --- | --- |
| **Supplementary Table 2. Characteristics of patients according to 30-day clinical outcome** | Page 3–4 |
| **Supplementary Table 3. Results of univariate analysis using Fine and Gray model** | Page 5–6 |
| **Supplementary Table 4. Results of internal validation using bootstrapping** | Page 7 |
| **Supplementary Table 5. Results of sensitivity analysis including only 'definite' cases as infection-attributable deaths** | Page 8 |
| **Supplementary Table 6. Results of sensitivity analysis using 90-day infection-attributable death as the outcome** | Page 9 |
| **Supplementary Table 7. Results of landmark analysis with day 3 as the index day, excluding cases who died before persistent bacteremia diagnosis** | Page 10 |
| **Supplementary Table 8. Results of multivariate Cox proportional hazards model for cause-specific hazard ratios in patients with S. aureus bacteremia** | Page 11 |
| **Supplementary Table 9. Results of subgroup analyses** | Page 12 |
| **Supplementary Figure 1. Schoenfeld residuals for proportional hazards assumption.** | Page 13 |
| **Supplementary Figure 2. Calibration plot for prediction 30-day attributable deaths in patients with SAB** | Page 14 |

**Supplementary Table 1. Microbiological characteristics of *S. aureus* isolated from patients**

| **Variables** | **Patients with SAB (N=1936)** |
| --- | --- |
| Vancomycin MIC by BMD method no. (%) |  |
| 0.5 | 35/1815 (1.9) |
| 1 | 1540/1815 (84.8) |
| 2 | 238/1815 (13.1) |
| 4 | 2/1815 (0.1) |
| *agr* dysfunction no. (%) | 823/1784 (46.1) |
| MLST type |  |
| ST72 | 479/1783 (26.9) |
| ST5 | 580/1783 (32.5) |
| Others | 724/1783 (40.6) |

MIC, minimal inhibitory concentration; BMD, broth micro-dilution; MLST, multi-locus sequence typing.

**Supplementary Table 2. Characteristics of patients according to 30-day clinical outcome**

| **Variables** | **Infection-attributable death  (N = 338)** | **Non-infection attributable death  (N = 106)** | **Survived  (N = 1492)** | **P** |
| --- | --- | --- | --- | --- |
| Age, median (IQR), years | 66 (56–73) | 64.5 (57–72) | 62 (51.5–71) | <0.001 |
| Male, no. (%) | 221 (65.4) | 62 (58.5) | 931 (62.4) | 0.34 |
| Mode of acquisition, no. (%) |  |  |  | 0.09 |
| Community-acquired | 48 (14.2) | 15 (14.2) | 219 (14.7) |  |
| Health-care associated | 126 (37.3) | 27 (25.5) | 447 (30.0) |  |
| Nosocomial | 164 (48.5) | 64 (60.4) | 826 (55.4) |  |
| Comorbidities, no. (%) |  |  |  |  |
| Malignancy | 197 (58.3) | 72 (67.9) | 663 (44.4) | <0.001 |
| Diabetes mellitus | 86 (25.4) | 34 (32.1) | 472 (31.6) | 0.08 |
| Hypertension | 136 (40.2) | 45 (42.5) | 608 (40.8) | 0.84 |
| Chronic kidney disease | 39 (11.5) | 6 (5.7) | 230 (15.4) | 0.01 |
| Liver cirrhosis | 72 (21.3) | 20 (18.9) | 214 (14.3) | 0.004 |
| Chronic lung disease | 13 (3.8) | 3 (2.8) | 39 (2.6) | 0.49 |
| Ischemic heart disease | 36 (10.7) | 8 (7.5) | 129 (8.6) | 0.49 |
| Charlson comorbidity index, median (IQR) | 4 (2–6) | 4 (2–6) | 2 (1–4) | <0.001 |
| Predisposing factors, no. (%) |  |  |  |  |
| Neutropenia | 19 (5.6) | 5 (4.7) | 88 (5.9) | 0.87 |
| Immunosuppressant agent | 18 (5.3) | 4 (3.8) | 110 (7.4) | 0.26 |
| Corticosteroid use | 107 (31.7) | 34 (32.1) | 336 (22.5) | <0.001 |
| Indwelling prosthetic devices, no. (%) |  |  |  |  |
| Central venous catheter | 123 (36.4) | 47 (44.3) | 590 (39.5) | 0.26 |
| Cardiac implantable electronic device | 2 (0.6) | 1 (0.9) | 19 (1.3) | 0.54 |
| Prosthetic heart valve | 11 (3.3) | 2 (1.9) | 50 (3.4) | 0.71 |
| Vascular graft | 17 (5.0) | 3 (2.8) | 121 (8.1) | 0.02 |
| Orthopedic implant | 10 (3.0) | 2 (1.9) | 60 (4.0) | 0.37 |
| Severity of infection, no. (%) |  |  |  | <0.001 |
| No sepsis | 32 (9.5) | 17 (16.0) | 307 (20.6) |  |
| Sepsis | 188 (55.6) | 72 (67.9) | 1048 (70.2) |  |
| Septic shock | 118 (34.9) | 17 (16.0) | 137 (9.2) |  |
| Elevated C-reactive protein  (≥10 mg/dL), no. (%) | 215 (63.6) | 41 (38.7) | 678 (45.4) | <0.001 |
| Main focus of infection, no. (%) |  |  |  |  |
| CVC related | 67 (19.8) | 29 (27.4) | 398 (26.7) | 0.03 |
| Peripheral catheter-related | 15 (4.4) | 6 (5.7) | 106 (7.1) | 0.17 |
| Pneumonia | 73 (21.6) | 14 (13.2) | 108 (7.2) | <0.001 |
| Skin and soft tissue infection | 17 (5.0) | 5 (4.7) | 149 (10.0) | 0.004 |
| Surgical wound infection | 16 (4.7) | 4 (3.8) | 87 (5.8) | 0.53 |
| Endocarditis | 17 (5.0) | 4 (3.8) | 51 (3.4) | 0.40 |
| Bone and joint infection | 24 (7.1) | 2 (1.9) | 135 (9.0) | 0.02 |
| Primary bacteremia | 76 (22.5) | 23 (21.7) | 235 (15.8) | 0.01 |
| Others | 36 (10.7) | 18 (17.0) | 223 (14.9) | 0.08 |
| Length of bacteremia, median (IQR), days | 3 (1–5) | 1 (0–1.5) | 1 (0–3) | <0.001 |
| Persistent bacteremia (≥3 days), no. (%) | 88 (40.9) | 24 (24.0) | 392 (26.5) | <0.001 |
| Time to appropriate antibiotic therapy, median (IQR), days |  |  |  |  |
| Length of antibiotic therapy, median (IQR) days | 6 (2–13) | 16 (11–21) | 23 (15–40) | <0.001 |
| Type of antibiotic therapy |  |  |  | <0.001 |
| Anti-staphylococcal beta-lactams | 73 (21.6) | 25 (23.6) | 594 (39.8) |  |
| Glycopeptides | 159 (47.0) | 60 (56.6) | 696 (46.6) |  |
| Others | 106 (31.4) | 21 (19.8) | 202 (13.5) |  |

IQR, interquartile range; CVC, central venous catheter.

**Supplementary Table 3. Results of univariate analysis using Fine and Gray model**

|  | **Infection-attributable mortality** | | **Non-infection-attributable mortality** | |
| --- | --- | --- | --- | --- |
| **Variable** | **sHR (95% CI)** | **P** | **sHR (95% CI)** | **P** |
| Age (per 10 y increase in age) | 1.17 (1.08–1.26) | **<0.001** | 1.12 (1.00–1.26) | **0.049** |
| Male | 1.14 (0.92–1.43) | 0.24 | 0.84 (0.57–1.24) | 0.38 |
| Mode of acquisition |  |  |  |  |
| Community-acquired | (reference) |  | (reference) |  |
| Healthcare-associated | 1.23 (0.88-1.72) | 0.23 | 0.83 (0.44-1.55) | 0.56 |
| Nosocomial | 0.88 (0.64-1.22) | 0.44 | 1.14 (0.65-2.00) | 0.64 |
| Malignancy | 1.57 (1.27–1.95) | **<0.001** | 2.34 (1.55–3.51) | **<0.001** |
| Diabetes | 0.76 (0.59–0.97) | **0.03** | 1.07 (0.72–1.61) | 0.73 |
| Hypertension | 0.98 (0.79–1.22) | 0.88 | 1.08 (0.73–1.58) | 0.71 |
| Chronic kidney disease | 0.77 (0.55–1.07) | 0.12 | 0.36 (0.16–0.81) | **0.01** |
| Liver cirrhosis | 1.49 (1.15–1.93) | **0.002** | 1.25 (0.77–2.04) | 0.36 |
| Chronic lung disease | 1.40 (0.81–2.40) | 0.23 | 0.98 (0.32–3.04) | 0.97 |
| Ischemic heart disease | 1.22 (0.87–1.70) | 0.26 | 0.83 (0.40–1.71) | 0.61 |
| Neutropenia | 0.96 (0.61–1.52) | 0.87 | 0.80 (0.33–1.99) | 0.64 |
| Immunosuppressant agent | 0.75 (0.47–1.21) | 0.24 | 0.52 (0.19–1.41) | 0.20 |
| Corticosteroid use | 1.45 (1.16–1.83) | **0.001** | 1.45 (0.97–2.18) | 0.07 |
| Central venous catheter | 0.87 (0.70–1.08) | 0.21 | 1.23 (0.84–1.80) | 0.29 |
| Cardiac implantable electronic device | 0.47 (0.13–1.79) | 0.27 | 0.82 (0.11–5.99) | 0.85 |
| Prosthetic heart valve | 0.98 (0.55–1.77) | 0.95 | 0.55 (0.14–2.21) | 0.40 |
| Vascular graft | 0.65 (0.40–1.05) | 0.08 | 0.37 (0.12–1.16) | 0.09 |
| Orthopedic implant | 0.77 (0.41–1.45) | 0.42 | 0.48 (0.12–1.92) | 0.30 |
| Severity of infection |  |  |  |  |
| No sepsis | (reference) |  | (reference) |  |
| Sepsis | 1.63 (1.12-2.36) | **0.01** | 1.14 (0.67-1.94) | 0.62 |
| Septic shock | 6.34 (4.29-9.37) | **<0.001** | 1.31 (0.67-2.57) | 0.43 |
| C–reactive protein ≥ 10 mg/dL | 2.00 (1.61-2.50) | **<0.001** | 0.66 (0.45-0.98) | **0.04** |
| Central venous catheter-related | 0.69 (0.53-0.90) | **0.01** | 1.09 (0.72-1.68) | 0.68 |
| Peripheral catheter-related | 0.64 (0.38-1.07) | 0.09 | 0.86 (0.38-1.97) | 0.72 |
| Pneumonia | 2.89 (2.22-3.75) | **<0.001** | 1.37 (0.78-2.40) | 0.27 |
| Skin and soft tissue infection | 0.51 (0.32-0.83) | **0.01** | 0.50 (0.21-1.23) | 0.13 |
| Surgical wound infection | 0.84 (0.51-1.39) | 0.51 | 0.66 (0.25-1.78) | 0.41 |
| Endocarditis | 1.43 (0.88-2.32) | 0.15 | 1.01 (0.38-2.69) | 0.99 |
| Bone and joint infection | 0.83 (0.55-1.25) | 0.37 | 0.21 (0.05-0.86) | **0.03** |
| Primary bacteremia | 1.48 (1.14-1.91) | **0.003** | 1.34 (0.85-2.12) | 0.21 |
| Persistent bacteremia | 1.81 (1.38-2.37) | **<0.001** | 0.80 (0.50-1.26) | 0.33 |
| Focus removal |  |  |  |  |
| Complete | (reference) |  | (reference) |  |
| Not complete | 2.81 (1.84-4.31) | **<0.001** | 1.20 (0.51-2.82) | 0.67 |
| No eradicable focus | 2.59 (2.03-3.31) | **<0.001** | 1.43 (0.96-2.14) | 0.08 |
| MRSA | 1.17 (0.95–1.45) | 0.14 | 1.50 (1.01–2.22) | **0.042** |
| Vancomycin MIC ≥ 2 | 0.74 (0.52-1.06) | 0.10 | 1.67 (1.12-2.49) | 0.55 |
| agr dysfunction | 1.21 (0.92-1.58) | 0.18 | 1.88 (1.27-2.79) | 0.01 |
| MLST type |  |  |  |  |
| ST72 | (reference) |  | (reference) |  |
| ST5 | 1.28 (0.91-1.80) | 0.15 | 2.73 (1.56-4.79) | **<0.001** |
| Others | 0.78 (0.54-1.11) | 0.16 | 1.27 (0.69-2.32) | 0.44 |

sHR, subdistribution hazard ratio; CI, confidence interval; MRSA, methicillin-resistant *S. aureus*; MSSA, methicillin-susceptible *S. aureus*; MIC, minimal inhibitory concentration; MLST, multi-locus sequence typing. Significant P values are indicated in bold

**Supplementary Table 4. Results of internal validation using bootstrapping**

| **Variables** | **sHR** | **Lower 95% CI** | **Upper 95% CI** |
| --- | --- | --- | --- |
| Age (per 10 y increase in age) | 1.14 | 1.03 | 1.27 |
| Malignancy | 1.54 | 1.14 | 2.06 |
| Liver cirrhosis | 2.15 | 1.54 | 3.01 |
| Corticosteroid use | 1.61 | 1.19 | 2.17 |
| Severity of infection |  |  |  |
| No sepsis | (reference) |  |  |
| Sepsis | 1.33 | 0.89 | 2.08 |
| Septic shock | 3.28 | 1.95 | 5.54 |
| Pneumonia | 1.81 | 1.18 | 2.70 |
| Persistent bacteremia | 1.73 | 1.29 | 2.33 |
| Focus removal |  |  |  |
| Complete | (reference) |  |  |
| Not complete | 2.40 | 1.26 | 3.99 |
| No eradicable focus | 1.49 | 1.10 | 2.06 |
| Elevated CRP | 1.60 | 1.18 | 2.19 |

sHR, subdistribution hazard ratio; CI, confidence interval; CRP, C-reactive protein.
The sHRs with 95 confidence intervals (CI) were estimated from a model that underwent 1,000 resampling iterations through bootstrapping.

**Supplementary Table 5. Results of sensitivity analysis including only 'definite' cases as infection-attributable deaths**

|  | **Attributable death** | | | **Non-attributable death** | | |
| --- | --- | --- | --- | --- | --- | --- |
| **Variable** | **sHR** | **95% CI** | **P** | **sHR** | **95% CI** | **P** |
| Age (per 10 y increase in age) | 1.15 | 1.01–1.31 | 0.03 | 1.14 | 1.03–1.28 | 0.02 |
| Malignancy | 1.69 | 1.23–2.34 | 0.001 | 1.70 | 1.22–2.37 | 0.002 |
| Liver cirrhosis | 1.81 | 1.20–2.73 | 0.01 | 1.70 | 1.15–2.52 | 0.01 |
| Corticosteroid use | 1.47 | 1.02–2.11 | 0.04 | 1.58 | 1.13–2.21 | 0.01 |
| Severity of infection |  |  |  |  |  |  |
| No sepsis |  |  |  |  |  |  |
| Sepsis | 1.04 | 0.63–1.71 | 0.88 | 1.57 | 0.97–2.53 | 0.06 |
| Septic shock | 3.40 | 1.94–5.96 | <0.001 | 1.99 | 1.09–3.62 | 0.03 |
| Pneumonia | 1.44 | 0.87–2.38 | 0.16 | 1.86 | 1.16–2.97 | 0.01 |
| Persistent bacteremia | 1.77 | 1.27–2.48 | <0.001 | 1.17 | 0.83–1.65 | 0.38 |
| Focus removal |  |  |  |  |  |  |
| Complete |  |  |  |  |  |  |
| Not complete | 2.38 | 1.24–4.57 | 0.01 | 1.55 | 0.80–3.01 | 0.19 |
| No eradicable focus | 1.58 | 1.09–2.29 | 0.02 | 1.30 | 0.92–1.83 | 0.14 |
| C-reactive protein ≥ 10 mg/dL | 1.52 | 1.08–2.15 | 0.02 | 0.94 | 0.67–1.31 | 0.70 |

sHR, subdistributional hazard ratio; CI, confidence interval; CRP, C-reactive protein.

**Supplementary Table 6. Results of sensitivity analysis using 90-day infection-attributable death as the outcome**

|  | **Attributable death** | | | **Non-attributable death** | | |
| --- | --- | --- | --- | --- | --- | --- |
| **Variable** | **sHR** | **95% CI** | **P** | **sHR** | **95% CI** | **P** |
| Age (per 10 y increase in age) | 1.19 | 1.08–1.31 | <0.001 | 1.15 | 1.05–1.25 | 0.002 |
| Malignancy | 1.49 | 1.16–1.91 | 0.002 | 2.06 | 1.58–2.69 | <0.001 |
| Liver cirrhosis | 1.93 | 1.41–2.63 | <0.001 | 1.61 | 1.19–2.17 | 0.002 |
| Corticosteroid use | 1.63 | 1.24–2.15 | <0.001 | 1.38 | 1.06–1.80 | 0.02 |
| Severity of infection |  |  |  |  |  |  |
| No sepsis |  |  |  |  |  |  |
| Sepsis | 1.32 | 0.90–1.95 | 0.16 | 1.19 | 0.86–1.67 | 0.30 |
| Septic shock | 3.08 | 1.94–4.87 | <0.001 | 1.54 | 0.99–2.42 | 0.06 |
| Pneumonia | 1.67 | 1.14–2.45 | 0.01 | 1.10 | 0.71–1.70 | 0.67 |
| Persistent bacteremia | 1.95 | 1.51–2.52 | <0.001 | 0.84 | 0.62–1.12 | 0.23 |
| Focus removal |  |  |  |  |  |  |
| Complete |  |  |  |  |  |  |
| Not complete | 2.77 | 1.73–4.44 | <0.001 | 1.32 | 0.78–2.23 | 0.30 |
| No eradicable focus | 1.50 | 1.13–1.99 | 0.01 | 1.10 | 0.85–1.43 | 0.46 |
| C-reactive protein ≥ 10 mg/dL | 1.57 | 1.21–2.05 | <0.001 | 0.81 | 0.62–1.06 | 0.12 |

sHR, subdistributional hazard ratio; CI, confidence interval; CRP, C-reactive protein.

**Supplementary Table 7. Results of landmark analysis with day 3 as the index day, excluding cases who died before persistent bacteremia diagnosis**

|  | **Attributable death** | | | **Non-attributable death** | | |
| --- | --- | --- | --- | --- | --- | --- |
| **Variable** | **sHR** | **95% CI** | **P** | **sHR** | **95% CI** | **P** |
| Age (per 10 y increase in age) | 1.14 | 1.02–1.26 | 0.02 | 1.17 | 1.02–1.34 | 0.02 |
| Malignancy | 1.52 | 1.16–2.00 | 0.003 | 2.19 | 1.42–3.37 | <0.001 |
| Liver cirrhosis | 2.16 | 1.56–2.99 | <0.001 | 1.16 | 0.69–1.95 | 0.58 |
| Corticosteroid use | 1.63 | 1.21–2.20 | 0.001 | 1.37 | 0.90–2.09 | 0.14 |
| Severity of infection |  |  |  |  |  |  |
| No sepsis |  |  |  |  |  |  |
| Sepsis | 1.33 | 0.86–2.05 | 0.20 | 1.23 | 0.70–2.15 | 0.47 |
| Septic shock | 3.22 | 1.94–5.34 | <0.001 | 1.83 | 0.90–3.72 | 0.10 |
| Pneumonia | 1.78 | 1.18–2.67 | 0.01 | 1.34 | 0.72–2.48 | 0.36 |
| Persistent bacteremia | 1.74 | 1.31–2.31 | <0.001 | 0.91 | 0.57–1.45 | 0.68 |
| Focus removal |  |  |  |  |  |  |
| Complete |  |  |  |  |  |  |
| Not complete | 2.41 | 1.38–4.19 | 0.002 | 1.18 | 0.47–2.97 | 0.72 |
| No eradicable focus | 1.49 | 1.08–2.04 | 0.01 | 1.32 | 0.86–2.01 | 0.20 |
| C-reactive protein ≥ 10 mg/dL | 1.59 | 1.19–2.13 | 0.002 | 0.66 | 0.42–1.02 | 0.06 |

sHR, subdistributional hazard ratio; CI, confidence interval; CRP, C-reactive protein.

**Supplementary Table 8. Results of multivariate Cox proportional hazards model for cause-specific hazard ratios in patients with *S. aureus* bacteremia**

|  | **Attributable death** | | | **Non-attributable death** | | |
| --- | --- | --- | --- | --- | --- | --- |
| **Variable** | **csHR** | **95% CI** | **P** | **csHR** | **95% CI** | **P** |
| Age (per 10 y increase in age)* | 1.14 | 1.03 - 1.26 | 0.01 | 1.20 | 1.03 - 1.39 | 0.02 |
| Malignancy | 1.56 | 1.18 - 2.06 | 0.002 | 2.36 | 1.55 - 3.61 | <0.001 |
| Liver cirrhosis | 2.16 | 1.54 - 3.02 | <0.001 | 1.28 | 0.76 - 2.17 | 0.35 |
| Corticosteroid use | 1.62 | 1.21 - 2.17 | 0.001 | 1.45 | 0.94 - 2.23 | 0.09 |
| Severity of infection |  |  |  |  |  |  |
| No sepsis |  |  |  |  |  |  |
| Sepsis | 1.34 | 0.87 - 2.06 | 0.19 | 1.27 | 0.72 - 2.23 | 0.41 |
| Septic shock | 3.35 | 2.06 - 5.44 | <0.001 | 2.26 | 1.09 - 4.66 | 0.03 |
| Pneumonia | 1.84 | 1.24 - 2.73 | 0.002 | 1.53 | 0.81 - 2.88 | 0.19 |
| Persistent bacteremia | 1.72 | 1.30 - 2.29 | <0.001 | 0.93 | 0.58 - 1.50 | 0.78 |
| Focus removal |  |  |  |  |  |  |
| Complete |  |  |  |  |  |  |
| Not complete | 2.43 | 1.41 - 4.20 | 0.001 | 1.36 | 0.53 - 3.47 | 0.53 |
| No eradicable focus | 1.50 | 1.09 - 2.05 | 0.01 | 1.40 | 0.90 - 2.16 | 0.13 |
| C-reactive protein ≥ 10 mg/dL | 1.59 | 1.18 - 2.14 | 0.002 | 0.71 | 0.46 - 1.08 | 0.11 |

csHR, cause-specific hazard ratio; CI, confidence interval; CRP, C-reactive protein.

**Supplementary** **Table 9. Results of subgroup analyses**

|  | **Age group** | |  | **Sex** | |  | **Mode of acquisition** | |  |
| --- | --- | --- | --- | --- | --- | --- | --- | --- | --- |
|  | **Age ≥ 60** | **Age < 60** |  | **Female** | **Male** |  | **Community-acquired** | **Non-community-acquired**^*^ |  |
| **Variables** | **sHR (95% CI)** | **sHR (95% CI)** | **P**^†^ | **sHR (95% CI)** | **sHR (95% CI)** | **P**^†^ | **sHR (95% CI)** | **sHR (95% CI)** | **P**^†^ |
| Age per 10 y increase | 1.15 (0.87, 1.52) | 1.10 (0.87, 1.39) | 0.67 | 1.06 (0.90, 1.24) | 1.20 (1.05, 1.37) | 0.10 | 1.12 (0.81, 1.53) | 1.13 (1.02, 1.26) | 0.09 |
| Malignancy | 1.49 (1.05, 2.13) | 1.73 (1.09, 2.74) | 0.61 | 1.69 (1.09, 2.62) | 1.51 (1.06, 2.14) | 0.67 | 1.41 (0.48, 4.12) | 1.44 (1.08, 1.93) | 0.88 |
| Liver cirrhosis | 2.33 (1.51, 3.60) | 1.98 (1.19, 3.29) | 0.64 | 2.25 (1.21, 4.17) | 2.20 (1.50, 3.25) | 0.96 | 1.36 (0.40, 4.61) | 2.21 (1.58, 3.09) | 0.37 |
| Corticosteroid use | 1.51 (1.02, 2.22) | 1.76 (1.07, 2.89) | 0.61 | 1.28 (0.79, 2.08) | 1.89 (1.28, 2.80) | 0.23 | 0.36 (0.03, 3.81) | 1.63 (1.20, 2.22) | 0.19 |
| Severity of infection |  |  |  |  |  |  |  |  |  |
| No sepsis | (reference) | (reference) |  | (reference) | (reference) |  | (reference) | (reference) |  |
| Sepsis | 1.47 (0.83, 2.59) | 1.22 (0.61, 2.45) | 0.70 | 2.31 (0.92, 5.80) | 1.05 (0.64, 1.73) | 0.08 | 4.57 (0.23, 92.44) | 1.24 (0.80, 1.94) | 0.60 |
| Septic shock | 3.83 (1.99, 7.37) | 2.73 (1.21, 6.12) | 0.53 | 5.78 (2.07, 16.13) | 2.67 (1.48, 4.84) | 0.15 | 17.02 (0.69, 417.88) | 3.03 (1.79, 5.13) | 0.39 |
| Pneumonia | 2.05 (1.27, 3.31) | 1.32 (0.60, 2.90) | 0.35 | 2.10 (0.98, 4.50) | 1.71 (1.03, 2.84) | 0.67 | 2.64 (0.61, 11.45) | 1.71 (1.12, 2.62) | 0.51 |
| Persistent bacteremia | 1.32 (0.90, 1.92) | 2.58 (1.66, 4.00) | 0.02 | 2.65 (1.64, 4.29) | 1.34 (0.93, 1.92) | 0.03 | 2.98 (0.76, 11.75) | 1.70 (1.26, 2.29) | 0.46 |
| Focus removal |  |  |  |  |  |  |  |  |  |
| Complete | (reference) | (reference) |  | (reference) | (reference) |  | (reference) | (reference) |  |
| Not complete | 2.42 (1.15, 5.10) | 2.47 (1.07, 5.69) | 0.96 | 1.45 (0.51, 4.10) | 3.04 (1.56, 5.95) | 0.24 | 61.48 (1.55, 2442.49) | 2.18 (1.23, 3.85) | 0.11 |
| No eradicable focus | 1.39 (0.92, 2.11) | 1.70 (1.02, 2.83) | 0.53 | 1.49 (0.91, 2.45) | 1.47 (0.97, 2.21) | 0.94 | 7.27 (0.78, 67.52) | 1.49 (1.08, 2.08) | 0.17 |
| Elevated CRP | 1.72 (1.18, 2.51) | 1.45 (0.90, 2.34) | 0.59 | 1.46 (0.88, 2.41) | 1.77 (1.22, 2.58) | 0.54 | 1.10 (0.27, 4.40) | 1.71 (1.27, 2.31) | 0.36 |

sHR, subdistributional hazard ratio; CI, confidence interval.

^*^Non-community-acquired includes healthcare-associated and nosocomial infections.

^†^P-value in table indicates P for interaction.

**Supplementary Figure 1. Schoenfeld residuals for proportional hazards assumption.**
Schoenfeld residuals for each variable in the univariate Cox regression model. The residuals are plotted against time to assess the proportional hazards assumption. None of the variables showed significant deviation from proportionality (P value > 0.05). The solid line represents the smoothed residuals, and the dashed lines indicate the 95 confidence intervals.


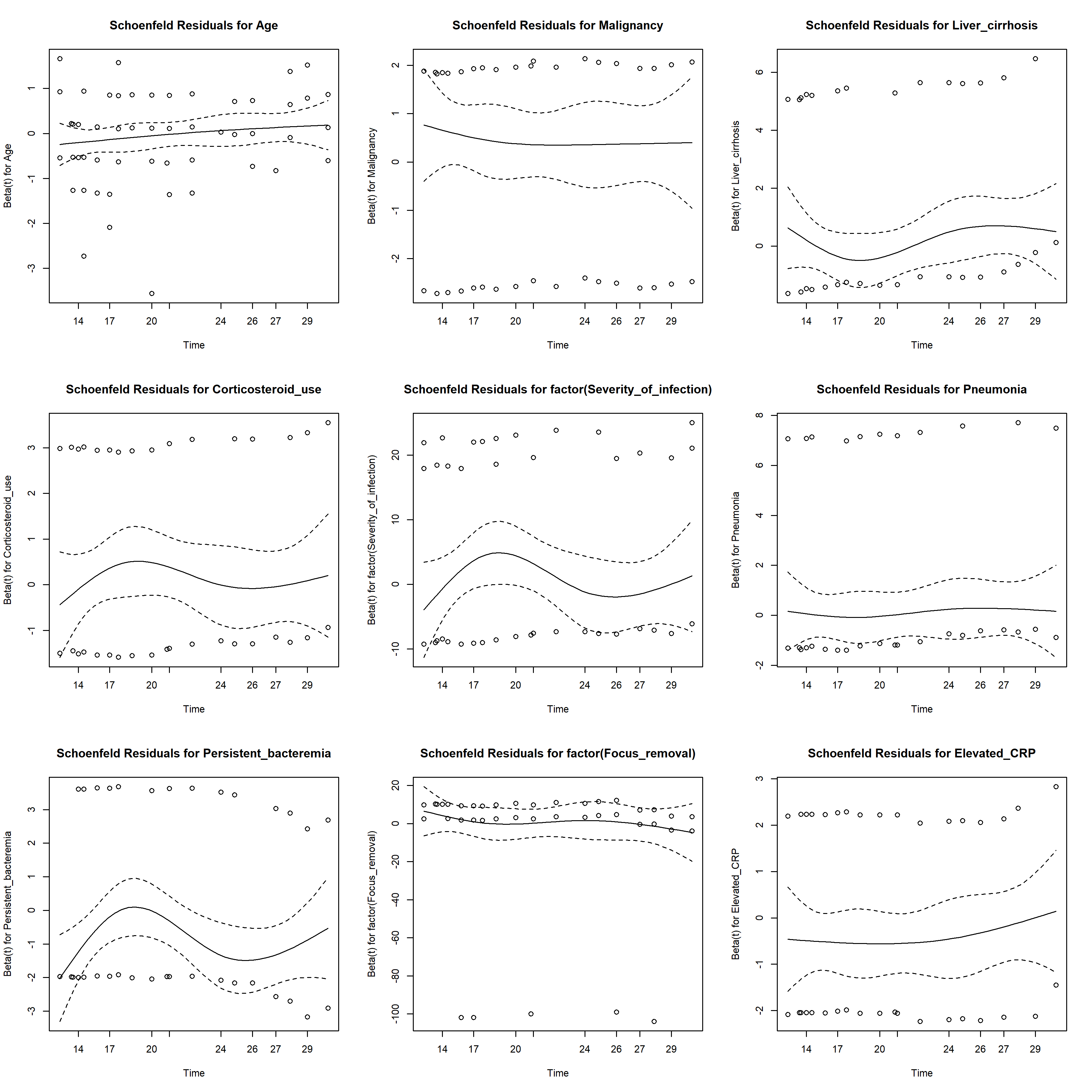


**Supplementary Figure 2.** **Calibration plot for prediction 30-day attributable deaths in patients with SAB.**
The calibration plot shows the agreement between the predicted risks from the attributable mortality risk prediction model and the observed outcome proportions. The 45-degree gray line represents perfect calibration. The solid black line indicates the calibration curve, reflecting the proportion of observed outcomes at each level of predicted risk. Censored data in the calibration curve were estimated by pseudo-observation. The black bars at the bottom represent the distribution of predicted risks among the patients. The intercept and slope of the calibration plot are 0.08 (0.04 to 0.12) and 1.26 (1.01 to 1.50), respectively.


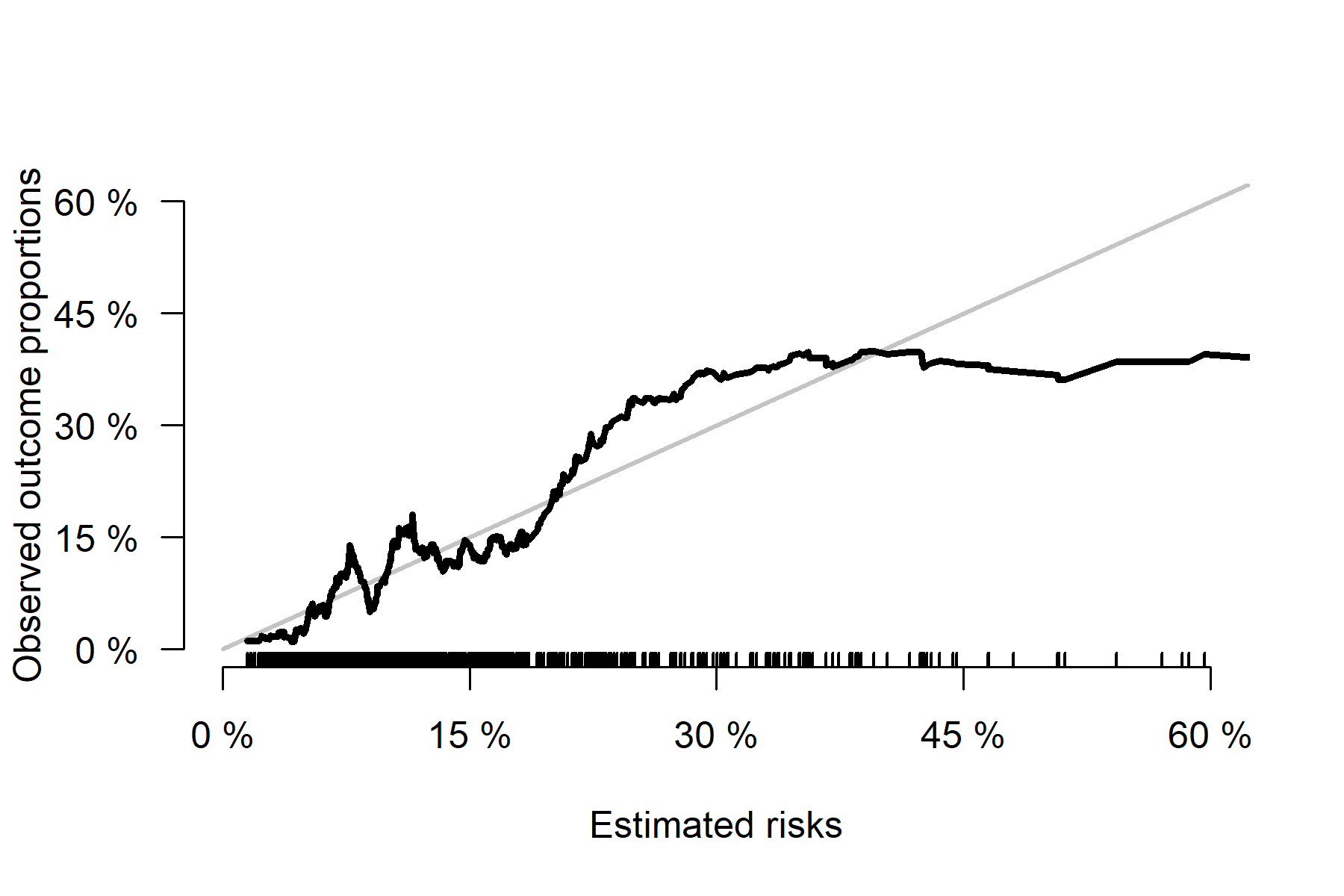

Supplement: ofae734_Supplementary_Data [file ofae734_supplementary_data.docx]
